# Clinical and Histopathologic Features of Interstitial Lung Disease in Erdheim–Chester Disease

**DOI:** 10.3390/jcm7090243

**Published:** 2018-08-28

**Authors:** Sara G. Haroutunian, Kevin J. O’Brien, Juvianee I. Estrada-Veras, Jianhua Yao, Louisa C. Boyd, Kavya Mathur, William A. Gahl, S. Mojdeh Mirmomen, Ashkan A. Malayeri, David E. Kleiner, Elaine S. Jaffe, Bernadette R. Gochuico

**Affiliations:** 1 Medical Genetics Branch, National Human Genome Research Institute, National Institutes of Health, Bethesda, MD 20892, USA; sharoutunian@gmail.com (S.G.H.); juvianee.estradaveras@nih.gov (J.I.E.-V.); lboyd6@uthsc.edu (L.C.B.); gahlw@mail.nih.gov (W.A.G.); 2 Office of the Clinical Director, National Human Genome Research Institute, National Institutes of Health, Bethesda, MD 20892, USA; obrienke@mail.nih.gov (K.J.O.); kavyamathur929@gmail.com (K.M.); 3 Radiology and Imaging Sciences, Warren Grant Magnuson Clinical Center, National Institutes of Health, Bethesda, MD 20892, USA; jianhua.yao@gmail.com; 4 Laboratory of Diagnostic Radiology Research, Warren Grant Magnuson Clinical Center, National Institutes of Health, Bethesda, MD 20892, USA; mojdeh.mirmomen@nih.gov (S.M.M.); amalayeri@bwh.harvard.edu (A.A.M.); 5 Laboratory of Pathology, Center for Cancer Research, National Cancer Institute, National Institutes of Health, Bethesda, MD 20892, USA; kleinerd@mail.nih.gov (D.E.K.); ejaffe@mail.nih.gov (E.S.J.)

**Keywords:** Erdheim–Chester disease, Factor XIIIa, interstitial lung disease, non-Langerhans cell histiocytosis, pulmonary fibrosis

## Abstract

Limited information is available regarding interstitial lung disease (ILD) in Erdheim–Chester disease (ECD), a rare multisystemic non-Langerhans cell histiocytosis. Sixty-two biopsy-confirmed ECD patients were divided into those with no ILD (19.5%), minimal ILD (32%), mild ILD (29%), and moderate/severe ILD (19.5%), based on computed tomography (CT) findings. Dyspnea affected at least half of the patients with mild or moderate/severe ILD. Diffusion capacity was significantly reduced in ECD patients with minimal ILD. Disease severity was inversely correlated with pulmonary function measurements; no correlation with *BRAF* V600E mutation status was seen. Reticulations and ground-glass opacities were the predominant findings on CT images. Automated CT scores were significantly higher in patients with moderate/severe ILD, compared to those in other groups. Immunostaining of lung biopsies was consistent with ECD. Histopathology findings included subpleural and septal fibrosis, with areas of interspersed normal lung, diffuse interstitial fibrosis, histiocytes with foamy cytoplasm embedded in fibrosis, lymphoid aggregates, and focal type II alveolar cell hyperplasia. In conclusion, ILD of varying severity may affect a high proportion of ECD patients. Histopathology features of ILD in ECD can mimic interstitial fibrosis patterns observed in idiopathic ILD.

## 1. Introduction

Erdheim–Chester disease (ECD) is a rare, multisystemic, non-Langerhans cell histiocytic neoplasm of unknown etiology [1,2,3,4]. Histopathologic features of ECD include foamy or granular histiocytes with well-defined borders, Touton-type giant cells, lymphocytes, and scattered plasma cells with surrounding fibrosis [5]. Lesional cells are generally positive for cluster of differentiation (CD) markers CD68, CD163, and factor XIIIa, and negative for CD1a and Langerin. S-100 is variably positive, and ultrastructural studies show absent Birbeck granules. Accumulation of ECD histiocytes results in chronic inflammation, fibrosis, and organ dysfunction [5]. A *BRAF* V600E mutation has been found in ECD and Langerhans cell histiocytosis, and the presence of this mutation helps differentiate the histiocytosis [6,7].

ECD has a clinically diverse phenotype, and treatment efficacy varies [6,8]. Consensus guidelines are available for empirical management of ECD [9]. Discovery of mutations in the mitogen-activated protein kinase pathway led to targeted treatment, including vemurafenib, a BRAF inhibitor, which is approved for patients harboring the *BRAF* mutation [10]. 

ECD patients may be affected by pulmonary disease, which is associated with poor survival [4,8]. Pleural disease and lung parenchymal involvement with interstitial lung disease (ILD) have been reported [11,12,13,14,15]. This study focuses on defining the ILD of ECD in a cohort of 62 patients. Baseline clinical findings, radiographic images, and physiological tests were studied. Chest imaging and the corresponding histopathological findings of ILD were analyzed in three patients.

## 2. Experimental Section

### 2.1. Patients and Molecular Studies

Written informed consent was obtained from 62 biopsy-confirmed ECD patients, who enrolled in protocol 11-HG-0207 (Clinical Trials NCT01417520, “Clinical and Basic Investigations into Erdheim–Chester disease”). The study was approved by the institutional review board of the National Human Genome Research Institute. Criteria for diagnosis of ECD and molecular testing for the *BRAF* V600E mutation were previously reported [4]. 

### 2.2. Pulmonary Physiology Testing

Six-minute walk tests and pulmonary function measurements were performed in accordance with American Thoracic Society/European Respiratory Society standards, as described (Vmax Encore PFT System, Vyaire, Yorba Linda, CA, USA) [16]. Single-breath diffusion capacity (DLCO) values were corrected for hemoglobin, but not lung volume.

### 2.3. Radiologic Imaging

Computed tomography (CT) scans of the chest were performed, and images were reviewed as described [15]. Patients were grouped into those with no ILD or with minimal, mild, or moderate/severe ILD by an expert in pulmonary fibrosis (BRG), as described [17,18]. High-resolution CT (HRCT) scans were analyzed by an automated quantification computer program previously validated in cohorts at risk for developing ILD and scored independently by an experienced imaging sciences investigator [18]. Reviewers were aware of patients’ ECD diagnoses, but were blinded to their clinical data. 

### 2.4. Histopathology and Immunohistochemistry

Fixed lung tissue sections from three ECD patients with symptomatic lung disease were stained with hematoxylin and eosin. Immunostaining for CD68 (clone KP-1, Roche 790–2931, prediluted), CD163 (clone MRQ-26, Roche 760–4437, prediluted), factor XIIIa (clone AC-1A1, Roche 760–4387, prediluted), CD1a (clone EP3622, Roche 760–4437, prediluted), langerin (clone 12D6, Leica NCL-Langerin, 1:200), and S-100 (clone 15E2E2, BioGenex MU058-UC, 1:8000) was performed on a Ventana Benchmark Ultra immunostaining machine.

### 2.5. Statistical Analysis

Data are shown as the mean ± standard error of the mean. Unpaired Student’s *t*-test, ANOVA, Fisher’s exact test, and chi-squared test were used for comparisons (GraphPad Prism 5, GraphPad Software, San Diego, CA, USA). A *p*-value < 0.05 was considered significant. 

## 3. Results

### 3.1. Clinical Features 

**Figure 1:**
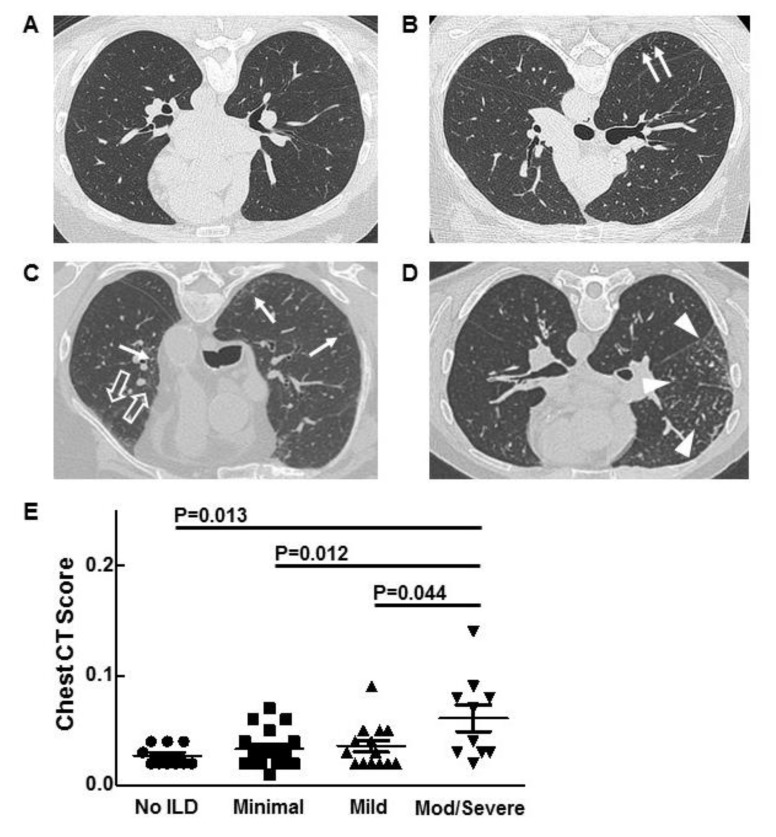
Representative high-resolution chest-computed tomography scan images from patients with Erdheim–Chester disease (ECD) and no interstitial lung disease (ILD) (**A**), minimal ILD with subpleural reticulations (arrows) (**B**), mild ILD with reticular ground-glass opacification (open arrow) (**C**), and moderate (mod) ILD with reticular nodular opacity (arrow heads) (**D**), are shown. Chest-computed tomography scan scores for patients with ECD and varying severity of ILD are displayed (**E**).

Sixty-two patients (45 males, 17 females) with ECD were evaluated for ILD. ECD affected at least one extrapulmonary site in all patients. Based on chest CT findings, 12 (19.5%) had no ILD, 20 (32%) had minimal ILD, 18 (29%) had mild ILD, and 12 (19.5%) had moderate/severe ILD (Figure 1A–D, Table 1). One patient with minimal ILD had a prior pneumonectomy to resect a tumor, which was interpreted as a paraganglioma. Mean ages were 51.8, 49.2, 55.5, and 55.8 years, respectively, for ECD patients without ILD, minimal ILD, mild ILD, and moderate/severe ILD.

Clinical staff asked patients about their pulmonary symptoms. Dyspnea was more common in ECD patients with mild or moderate/severe ILD (50.0% or 58.3%, respectively) compared to those with no ILD or minimal ILD (25%). Less than one-third of patients experienced chronic cough. No respiratory symptoms were reported in 58.3%, 65%, 38.9%, and 33%, respectively, of ECD patients with no ILD, minimal ILD, mild ILD, and moderate/severe ILD. Crackles were audible in 16.7% of ECD patients with mild or moderate/severe ILD.

Fifty (80.6%) of the 62 ECD patients never smoked, including more than 90% of patients with no ILD or minimal ILD. None of the ECD patients with no ILD or minimal ILD actively smoked, but 8.3% and 11.1% of ECD patients with mild ILD or moderate/severe ILD were current smokers. ECD patients with no ILD had no history of inhalational exposures; some ECD patients with ILD reported inhalational exposures, including herbicides, pesticides, and agricultural chemicals. Treatments for patients in our cohort included BRAF and MEK inhibitors (e.g., vemurafenib, dabrafenib, trametinib), site-directed external beam radiation, rituximab, interferon-alpha, corticosteroids, and/or traditional chemotherapy. The severity of ILD did not correlate with the BRAF mutation status.

Laboratory values revealed no significant differences in white blood cell count, hemoglobin, or erythrocyte sedimentation rate among the four groups of patients. Anti-nuclear antibody and rheumatoid factor levels were positive in some patients; no patient was diagnosed with an autoimmune disorder.

### 3.2. Pulmonary Function Measurements

Lung physiology measurements included forced vital capacity (FVC), forced expiratory volume in 1-second (FEV_1_), total lung capacity (TLC), DLCO, and six-minute walk distance (Table 2). ECD patients with no ILD had normal pulmonary function tests (i.e., values 80%–120% predicted). DLCO, but not FVC or TLC, was significantly lower in ECD patients with minimal ILD compared to those without ILD (*p* = 0.007). FVC and DLCO were significantly lower in ECD patients with mild ILD compared to those without ILD (*p* = 0.031 and *p* < 0.001, respectively), and TLC and DLCO were significantly lower in ECD patients with mild ILD compared to those with minimal ILD (*p* = 0.022 and *p* < 0.001, respectively). ECD patients with moderate/severe ILD had restriction and moderate reduction in diffusion capacity, and their values were significantly lower than those of ECD patients with no ILD or with minimal ILD. Measurements of FVC and FEV_1_ were significantly lower in ECD patients with moderate/severe ILD compared to those with mild ILD (*p* = 0.045 and *p* = 0.009, respectively) (FEV_1_. Fifty-one patients completed six-minute walk tests; some were unable to complete testing due to neurological involvement. Overall, ECD patients with no ILD walked further than those with ILD, and the distance walked progressively decreased with increasing severity of ILD in ECD patients. However, no statistically significant differences in total distance ambulated were observed between groups of patients (Table 2). 

### 3.3. Chest Imaging and Scoring

Reticulations and ground-glass opacities were predominant lung parenchymal findings on chest CT and HRCT scans. Reticulations were found in 70.9% of ECD patients. Focal reticulations were present in 41.9%, and diffuse reticulations were seen in 29%. Ground-glass opacification in a subpleural, perivascular, or diffuse distribution was identified in 41.9% of ECD patients. Both reticulations and ground-glass opacities were found in 32.3% of patients. Honeycombing was observed in 8% of patients.

HRCT scans were analyzed by a previously validated automated quantification computer program [18]. The scores for ECD patients with moderate/severe ILD were significantly higher than those of patients with no ILD, minimal ILD, and mild ILD (*p* = 0.013, *p* = 0.012, and *p* = 0.044, respectively) (Figure 1E). HRCT scan scores did not correlate with lung function.

### 3.4. Corresponding Radiographic and Histopathologic Findings

**Figure 2:**
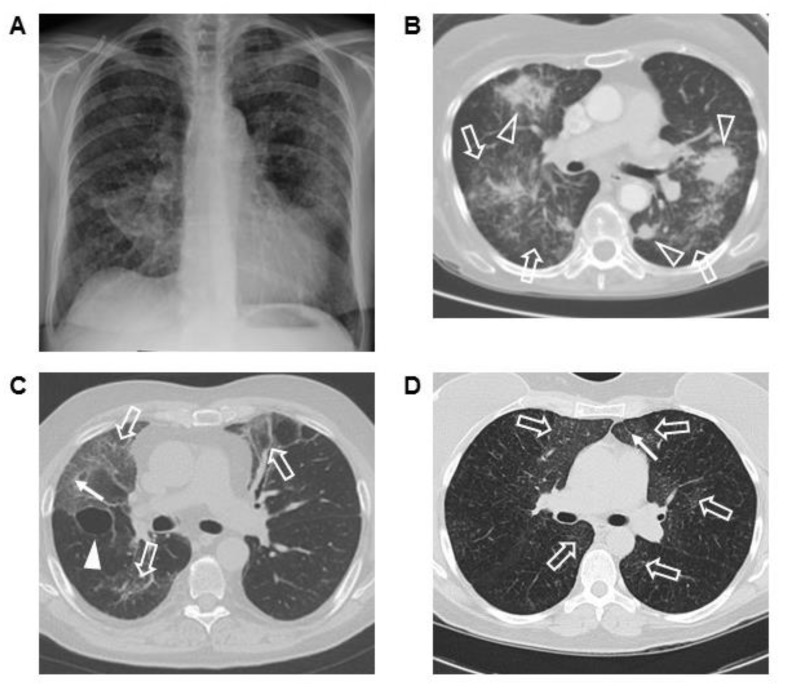
Posteroanterior chest radiograph (**A**) and representative computer tomography (CT) scan of the chest image (**B**) from a patient with Erdheim–Chester disease (ECD) and severe interstitial lung disease (ILD). Multiple bilateral consolidated masses (open arrowheads) with ground-glass infiltrates (open arrow) are found (**B**). Ground-glass infiltrates, reticulations (arrow), and a right lung cyst (solid arrowhead) are shown in another patient with ECD and severe ILD (**C**). Diffuse ground-glass opacification and reticulations are demonstrated in a third patient (**D**).

**Figure 3:**
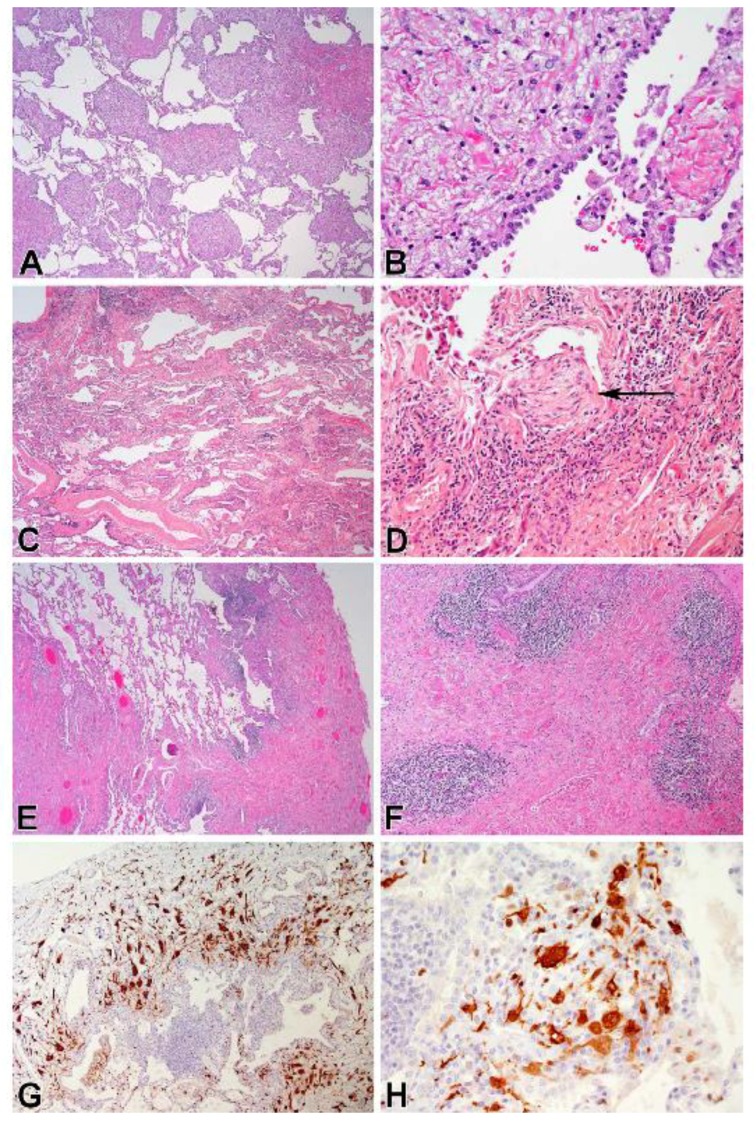
Representative pulmonary pathology images from a patient with Erdheim–Chester disease (ECD) show nodular areas of fibrosis with intervening normal alveolar parenchyma (**A**) (H&E, 40×) and type II pneumocyte hyperplasia overlying an area of fibrosis (**B**) (H&E, 400×). Photomicrographs of lung tissue from another ECD patient reveal diffuse interstitial fibrosis (**C**) (H&E, 40×) and a fibroblast focus (arrow) protruding from dense fibrosis with moderate lymphocytic inflammation (**D**) (H&E, 200×). Images from a third ECD patient demonstrate dense pleural and septal fibrosis with areas of normal alveolar parenchyma (**E**) (H&E, 40×), lymphoid aggregates within a region of dense fibrosis (**F**) (H&E, 100×), abnormal histiocytic infiltrate highlighted by immunostaining for factor XIIIa (**G**) (anti-factor XIIIa, 100×), and histocytes with abundant cytoplasm and slender cell processes (**H**) (anti-factor XIIIa, 400×).

Three ECD patients with severe ILD had chest imaging and lung biopsies. Chest radiograph and CT scan from one patient showed bilateral interstitial lung disease with ground-glass opacification, parenchymal masses, and airspace disease (Figure 2A,B). Sections of the open lung biopsy showed heterogeneous involvement of the lung parenchyma with normal areas interrupted by nodular fibrotic interstitial expansion lined by type II pneumocytes and infiltrated by histiocytes (Figure 3A,B). In addition to histiocytic infiltration, there was a mild lymphoplasmacytic infiltrate. The histiocytes stained with CD68, CD163, and S-100 and showed evidence of emperipolesis. Factor XIIIa, CD1a, and langerin were negative. 

Another ECD patient had bilateral ground-glass infiltrates as the primary radiographic finding; peripheral reticulations and a lung cyst were also seen (Figure 2C). Sections from the lung biopsy showed extensive interstitial fibrosis. In some areas, the fibrotic pattern mimicked a non-specific interstitial pneumonia pattern with uniform fibrotic widening of alveolar septa, while other areas showed a usual interstitial pneumonia pattern with temporally heterogeneous fibrosis and fibroblastic foci (Figure 3C,D). The interstitial inflammation varied from mild to moderate infiltrates of lymphocytes and plasma cells, and the fibrotic septa were covered with type II pneumocytes. Collections of pigmented alveolar macrophages filled alveolar spaces, while the interstitial histiocytes were stained with antibodies to CD68, CD163, and factor XIIIa. The histiocytes were negative for S-100 protein and CD1a. This patient developed progressive ILD and died due to respiratory insufficiency. 

The chest CT scan of a third patient with ECD and severe ILD showed diffuse ground-glass opacities and subpleural reticulations (Figure 2D). Histopathological evaluation of the open lung biopsy showed dense subpleural, peribronchial, and septal fibrosis, with intervening areas of normal lung (Figure 3E,F). Fibroblast foci were not apparent, but the fibrotic septa were lined by type II pneumocytes. The inflammatory infiltrate was sparse except for scattered lymphoid aggregates located adjacent to bronchioles or in the fibrotic areas. The histiocytes within the fibrosis stained diffusely for both CD163 and factor XIIIa (Figure 3G,H), and focally for S-100 protein. Langerin and CD1a were negative in the histiocytes, although both identified cells near the lymphoid aggregates. *BRAF* V600E was detected in sections from the lung biopsy.

## 4. Discussion

Erdheim–Chester disease is a rare multisystemic histiocytic disorder associated with ILD [4,5,8,11,12,13,14,15]. Chest CT scan can detect early, pre-clinical ILD in populations at risk of pulmonary fibrosis and in the general population [19,20,21,22]. Focal or diffuse reticulations and/or subpleural, perivascular, or diffuse ground-glass opacities were observed on CT scans in this ECD cohort. Approximately 80% of patients had reticulations or ground-glass opacifications to some degree, identified by chest CT scan imaging.

The mean age of our patients with or without ILD was similar; ILD generally affects middle-aged adults, which agrees with findings in an independent cohort of ECD patients [13]. These data are consistent with some disorders associated with ILD, such as rheumatoid arthritis and inherited conditions with high risk of fibrotic lung disease (e.g., familial pulmonary fibrosis, Hermansky–Pudlak syndrome pulmonary fibrosis) [17,19,20,23].

We examined potential risk factors for ILD in our patients with ECD. The percentage of patients with *BRAF* V600E mutations and positivity for anti-nuclear antibody or rheumatoid factor did not differ significantly among groups. One-fourth to one-third of ECD patients with mild or moderate/severe ILD were former or current smokers; in comparison, a smaller percentage (i.e., 8%) of ECD patients with no ILD had a history of smoking. Smoking is a risk factor for idiopathic pulmonary fibrosis, pre-clinical ILD in rheumatoid arthritis, and familial pulmonary fibrosis [17,20]. Our data indirectly suggest that smoking may also be a risk factor for ILD in ECD. Furthermore, a history of inhalational exposure to pesticides and other agricultural chemicals was identified in some ECD patients with ILD, and not in patients without ILD. Some ECD patients are treated with chemotherapeutic drugs associated with pulmonary toxicity. Although patients with and without ILD in this cohort received chemotherapeutic drugs, the possibility that medications may have contributed to ILD in some patients cannot be excluded.

Dyspnea, the predominant respiratory symptom, was reported by at least half of the patients with mild or moderate/severe disease. It is possible that some asymptomatic ECD patients with ILD are sedentary, due to concomitant extrapulmonary disease, and these patients with limited mobility might experience dyspnea with activity. It is interesting that one-fourth of patients with no ILD or minimal ILD also experienced dyspnea, indicating that dyspnea in some ECD patients is unrelated to their pulmonary disease. Dyspnea in these patients may be related to extrapulmonary involvement, such as cardiac disease.

Pulmonary function tests revealed restriction and impairment of gas exchange in ECD patients with moderate/severe ILD. Our results show that DLCO is more sensitive than FVC or TLC in detecting ILD, which agrees with previous findings in early familial pulmonary fibrosis [17]. DLCO measurements were normal in our ECD patients with no ILD, and values declined to below normal with increasing severity of ILD. FVC also decreased with worsening severity of lung disease, but only the group with moderate/severe ILD had measurements below normal.

ECD is a complex and potentially life-threatening disorder, and lung involvement is associated with poor survival [8]. Ninety percent of our patients had disease affecting multiple organs, and isolated pulmonary involvement was not seen in any patient with single organ disease [4]. However, lung disease may be the sole manifestation of ECD in other patients, and lung transplantation was performed in a patient with ECD and severe lung disease [24,25]. Furthermore, lung disease was a prominent manifestation in three patients in this cohort; one died of progressive respiratory insufficiency. Thus, this report provides evidence that although ILD may not be the leading cause of symptoms in most ECD patients, ILD can be an important cause of morbidity and mortality.

We compared imaging findings of three ECD patients with ILD with their lung histopathology. Their main radiographic finding was ground-glass infiltrates; lung masses were seen in one patient, and reticulations were observed in two. Immunohistochemical markers were consistent with ECD, and histiocytes were embedded in fibrotic regions. Histologic findings of ILD varied and included interstitial fibrosis that mimicked patterns observed in idiopathic forms of ILD. All three cases had areas of relatively normal alveolar parenchyma. Non-histiocytic inflammation was generally mild, with more intense infiltrates seen in one patient and multiple lymphoid aggregates seen in another. Pigmented alveolar macrophages were present in all cases and prominent in one.

## 5. Conclusions

Overall, this comprehensive analysis expands the phenotype of ILD in ECD. Many cases of ILD in ECD patients are asymptomatic, but can be diagnosed by CT scan. It is possible that ILD is underreported, because dyspnea, the most common respiratory symptom, may not develop in ECD patients due to activity limitations from extrapulmonary disease. Smoking may be a risk factor for ILD in ECD. DLCO is more sensitive than FVC or TLC in detecting ILD, and pulmonary function tests correlate inversely with the severity of ILD. Although isolated pulmonary involvement is uncommon, some ECD patients develop advanced ILD. Pathologic findings of ILD in ECD patients vary and may include subpleural and septal fibrosis, foamy histiocytes embedded in fibrotic regions, lymphoid aggregates, and focal type II alveolar cell hyperplasia. Further studies investigating pathogenic mechanisms of disease and treatment for ECD patients with ILD are indicated.

## Figures and Tables

**Table 1:** Clinical Characteristics of Patients with Erdheim–Chester Disease.

	No ILD	Minimal ILD	Mild ILD	Mod/Severe ILD
**Gender (Male/Female)**	9/3	15/5	14/4	7/5
**Age (years)**	51.8 ± 2.5	49.2 ± 2.5	55.5 ± 3.1	55.8 ± 3.4
**Dyspnea**	25%	25%	50%	58.3%
**Cough**	8.3%	15%	16.7%	33.3%
**No Symptoms**	58.3%	65%	38.9%	33%
**Crackles**	0%	0%	16.7%	16.7%
**Wheezing**	0%	0%	5.6%	16.7%
**Previous Smoker**	8.3%	10%	22.2%	16.7%
**Current Smoker**	0%	0%	11.1%	8.3%
**Inhalational Exposures**	0%	15%	5.6%	8.3%
**Chemotherapy**	75%	45%	50%	75%
**BRAF Inhibitor**	16.7%	10%	11.1%	16.7%
**MEK Inhibitor**	0%	5%	5.6%	0%
**Rituximab**	0%	5%	5.6%	8.3%
**Interferon**	33.3%	60%	50%	33.3%
***BRAF*** **V600E**	41.7%	60%	44.4%	58.3%
**ANA Positive**	33.3%	25%	22.2%	18.2%
**RF Positive**	8.3%	0%	11.1%	18.2%

ANA, anti-nuclear antibody; ILD, interstitial lung disease; Mod, moderate; RF, rheumatoid factor.

**Table 2:** Lung Function of Patients with Erdheim–Chester Disease.

	No ILD	Minimal ILD	Mild ILD	Mod/Severe ILD	*p*-Value
**FVC%**	98.6 ± 3.3	93.9 ± 3.2	85.1 ± 4.3	71.3 ± 4.7	0.345 * 0.031 ^†^ <0.001 ^‡^ 0.103 ** <0.001 ^††^ 0.045 ^‡‡^
**TLC%**	94.8 ± 2.7	96.5 ± 3.1	85.5 ± 3.4	74.5 ± 4.5	0.717 * 0.056 ^†^ <0.001 ^‡^ 0.022 ** <0.001 ^††^ 0.057 ^‡‡^
**DLCO%**	88.2 ± 2.9	77.2 ± 2.3	64.7 ± 2.0	58.2 ± 4.7	0.007 * <0.001 ^†^ <0.001 ^‡^ <0.001 ** <0.001 ^††^ 0.171 ^‡‡^
**6-MWT (m)**	504 ± 38	475 ± 19	432 ± 24	404 ± 37	0.461 * 0.106 ^†^ 0.077 ^‡^ 0.166 ** 0.070 ^††^ 0.521 ^‡‡^

6-MWT, six-minute walk test distance; DLCO%, diffusion capacity percent predicted; FVC%, forced vital capacity percent predicted; ILD, interstitial lung disease; mod, moderate; TLC%, total lung capacity percent predicted. * no ILD versus minimal ILD; ^†^ no ILD versus mild ILD; ^‡^ no ILD versus moderate/severe ILD; ** minimal ILD versus mild ILD; ^††^ minimal ILD versus moderate/severe ILD; ^‡‡^ mild ILD versus moderate/severe ILD.
